# Building a Simplistic Automatic Extruder: Instrument Development Opportunities for the Laboratory

**DOI:** 10.1021/acs.jchemed.4c00287

**Published:** 2024-08-01

**Authors:** Stefanie Klisch, Dylan Gilbert, Emma Breaux, Aliyah Dalier, Sudipta Gupta, Bruno Jakobi, Gerald J. Schneider

**Affiliations:** † Department of Chemistry, Louisiana State University, Baton Rouge, Louisiana 70803, United States; ‡ Department of Chemistry and Physics Southeastern Louisiana University, Hammond, Louisiana 70402, United States; § Department of Physics & Astronomy, Louisiana State University, Baton Rouge, Louisiana 70803, United States

**Keywords:** Upper-Division Undergraduate, Laboratory Instruction, Interdisciplinary, Hands-On Learning, Problem Solving, Laboratory Equipment, Lipids, Membranes

## Abstract

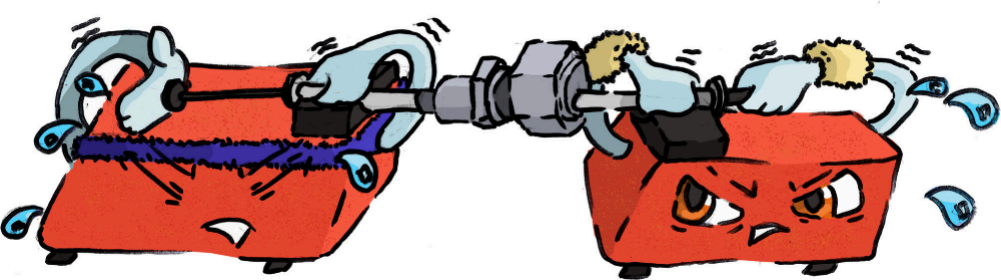

This work presents an automatic extruder as a research experience for undergraduate students. The system offers a user-friendly approach to preparing vesicles, such as liposomes or polymersomes, with a defined size and polydispersity—properties crucial for research in biology and macromolecules. It comprises two syringe pumps connected by a membrane filter. The setup is controlled by software. Compared to manual extrusion, this automated system provides advantages, such as precisely controlled variables. The project describes a tool to enhance undergraduate learning in science and engineering laboratories. Building an automatic extruder serves as a simplified model of a complex industrial process. It offers a clear advantage: automating a well-understood manual extrusion process. To make this project accessible, it is broken down into three manageable tasks: software development, hardware assembly, and testing procedures. This breakdown describes the software created, the hardware components used, and the testing procedures conducted for this project. All project data, including software code, testing data, and procedures, are freely available online. This allows undergraduate students to not only begin their own projects but also contribute to this educational instrument’s ongoing development.

## Introduction

Extrusion is a widely used technique for producing liposomes, polymersomes, and other scientific materials. However, achieving high-throughput and reliable extrusion over an extended period of time necessitates an automated extruder. This project introduces the development of open-source hardware and software for such an instrument. Its simplicity makes it ideal as a model to understand how complex industrial processes can be broken down into manageable modules. This project is perfect for students interested in various areas, including software development, hardware development, system benchmarking, or even formulating new scientific questions that can be addressed by implementing new software and hardware functionalities.

First described in 1964,^1^ liposomes have become an important asset to modern research. The scientific applications of liposomes are vast. The recent boom in gene therapies now takes advantage of the inner aqueous core of vesicle systems to transport medical cargo.^2^ Further applications include cosmetics,^3^ medical imaging,^4^ and vaccines.^5^

Liposomes have also made their way into teaching laboratories. The analysis of lipids found in everyday food items is a popular topic.^6^ Their properties can be demonstrated in a hands on or worksheet fashion.^7^ Del Bianco leads students to discover the dynamics of assembled lipids with a fluorescence-based project.^8^ Other authors focus on teaching students about transport across the bilayer membrane.^9^ Almendro et al. even teach students to prepare giant unilamellar vesicles using smartphones to build a simple *electroformation* device.^10^

The essential properties of liposomes are extensive and depend on parameters such as shape, diameter, and chemical composition. These properties can be measured using techniques, including electron microscopy, static and dynamic light scattering, small-angle X-ray scattering, and small-angle neutron scattering. In the following, the liposome diameter and diameter distribution become test parameters. They will be evaluated using dynamic light scattering because it can reliably and quickly determine the diameter and diameter distribution.

Research projects that take advantage of lipid vesicles begin with the preparation of liposomes. Several techniques are available, including electroporation,^11^ sonication,^12^ the temperature switch method,^11^ a caterpillar mixer,^13^ and the double emulsion technique.^14^ Due to the simplicity of use and the ability to control vesicle radius, extrusion has become a standard method.^12,15^ It relies purely on the physical passage of the vesicles through the pores of the membrane, without the need for additives that may change liposome properties.^16,17^

**Figure 1:**
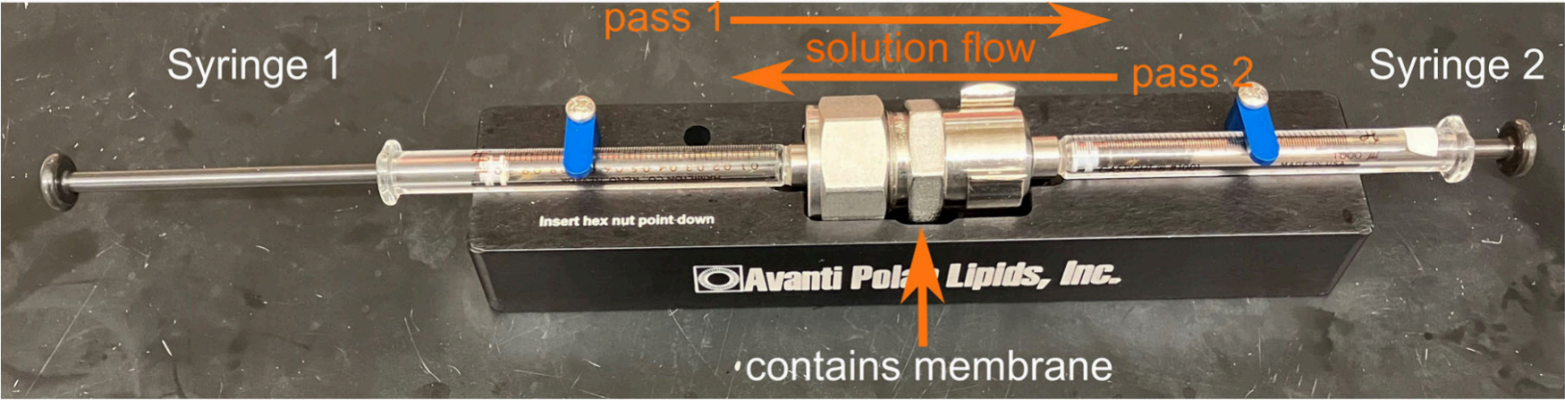
A manual extruder made up of a casing containing a membrane and its support structures, connected to two syringes. The syringes are pressed alternately in order to push the lipid solution back and forth through the membrane.

The extrusion process must provide materials of a specific diameter, which requires pressing a solution through a membrane filter multiple times. During manual extrusion, an experimentalist pushes the solution through the membrane by alternately pressing one of two syringes (Figure 1). This method is affordable and straightforward to learn. Simple hand extruders consist of two glass syringes, a membrane, and support structures.

Commercial tools like the Avanti Mini-extruder,^18^ the Liposofast Liposome Factory,^19^ the Twist by Helix Biotech,^20^ or the Genizer Hand Extruder^21^ are preconfigured sets that can be used to manually extrude amphiphilic molecules to obtain vesicles. Fifteen or more extrusion passes are standard to create specific size and size uniform liposomes.^22^ Hence, the preparation of multiple samples can be long, monotonous, and exhausting.

Automatic extrusion is an attractive alternative, and several possibilities are commercially available.^15,16,23−25^ However, commercial auto extruders are costly. Depending on the manufacturer, the software and hardware are proprietary and cannot be modified. In addition, they may require students to work with pressurized gas.^17,24−26^

**Figure 2:**
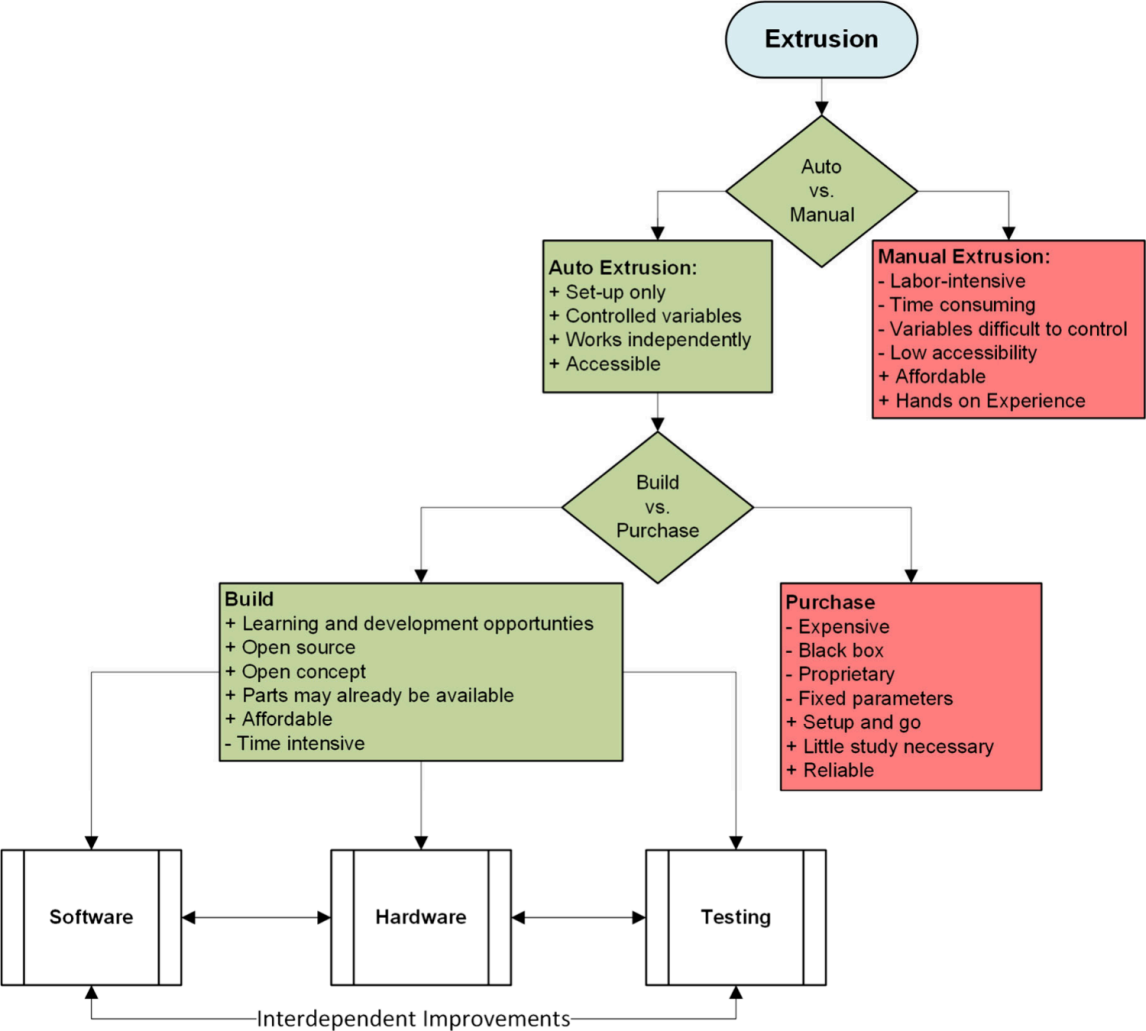
Flowchart illustrating the decision-making process for the acquisition of an in-lab extrusion system, suited to the author’s lab.

Building an extruder is especially useful for educational purposes because it is a straightforward process where each step is easily observable and adaptable to automation. It is a simplified model of a complex industrial process, allowing for the creation and optimization by introducing individual modules. In this case, these modules introduce students to the concepts of process design, software, hardware, and scientific principles. The focus is placed on reliably producing samples with specific diameters and a controlled diameter distribution. This reproducibility introduces the concept of a scientific need. The individual processes are interdependent. For example, extruding liposomes with a higher phase transition temperature necessitates elevated temperatures. This, in turn, requires the installation of a software-controlled heating mantle. Figure 2 shows the decisions made by the authors to build the automatic extruder presented here.

This work presents a procedure to build an automatic extruder using affordable parts that are often available in a typical chemistry laboratory. The software and hardware specifications are accessible and can be adapted as needed. Any interested group can copy the design and extend it as necessary. In accordance with the skill set of the students working on the project in the authors’ lab, LabVIEW was chosen as the most suitable programming tool. The supplied software can be used directly and adapted to suit another group’s needs. It can be downloaded for free from the authors’ GitHub page.^27^ The Web site offers a repository to everyone who wishes to contribute to the project. Finally, the assembly and testing of a minimalistic automatic extrusion system are described.

An interdisciplinary group of undergraduate students, supported by a graduate student, completed this project. Two undergraduate students developed the automatic extruder described hereafter during the summer. Student 1 assembled the two syringe pumps and programmed the software. Student 2 verified manually and automatically extruded the liposomes for comparison. A graduate student introduced the undergraduate students to the extrusion process and dynamic light scattering (DLS) instrument. Additionally, the graduate students independently verified the results. The process of building the automatic extruder is aimed at students interested in chemistry, mechanical engineering, and information technology. It contains individual work packages that are aimed at each student.

Verification includes comparing the results from the automatic extruder with those from manual extrusion. For that purpose 1,2-Dioleoyl-*sn-*glycero-3-phosphocholine (DOPC), a common,^28^ safe,^29^ and well-studied amphiphile was used. DOPC is known to form vesicles when prepared by extrusion. This lipid consists of a negatively charged phosphatidyl group, a positively charged choline, and two oleoyl tails.^28^ Because phosphatidylcholines remain zwitterionic over a wide pH range,^30^ their behavior is unlikely to be affected by small changes in pH.

The following text includes a description of the technical realization of the project as well as a verification of the work. The automatic extruder is shown to function reliably. The extrusion of self-assembled vesicular systems can be a part of the work in a macromolecular chemistry research group. It also demonstrates a process that is used in industry.^31^

## Materials and Methods

This section describes the procedure to make liposomes as well as the dynamic light scattering setup that was used to determine the size and size distribution. Links to Youtube videos explaining the extrusion process and introductions to dynamic light scattering have been added to the author’s GitHub page. Additional books and texts not referenced in this work are also listed there.

### Preparation of Liposomes

**Figure 3:**
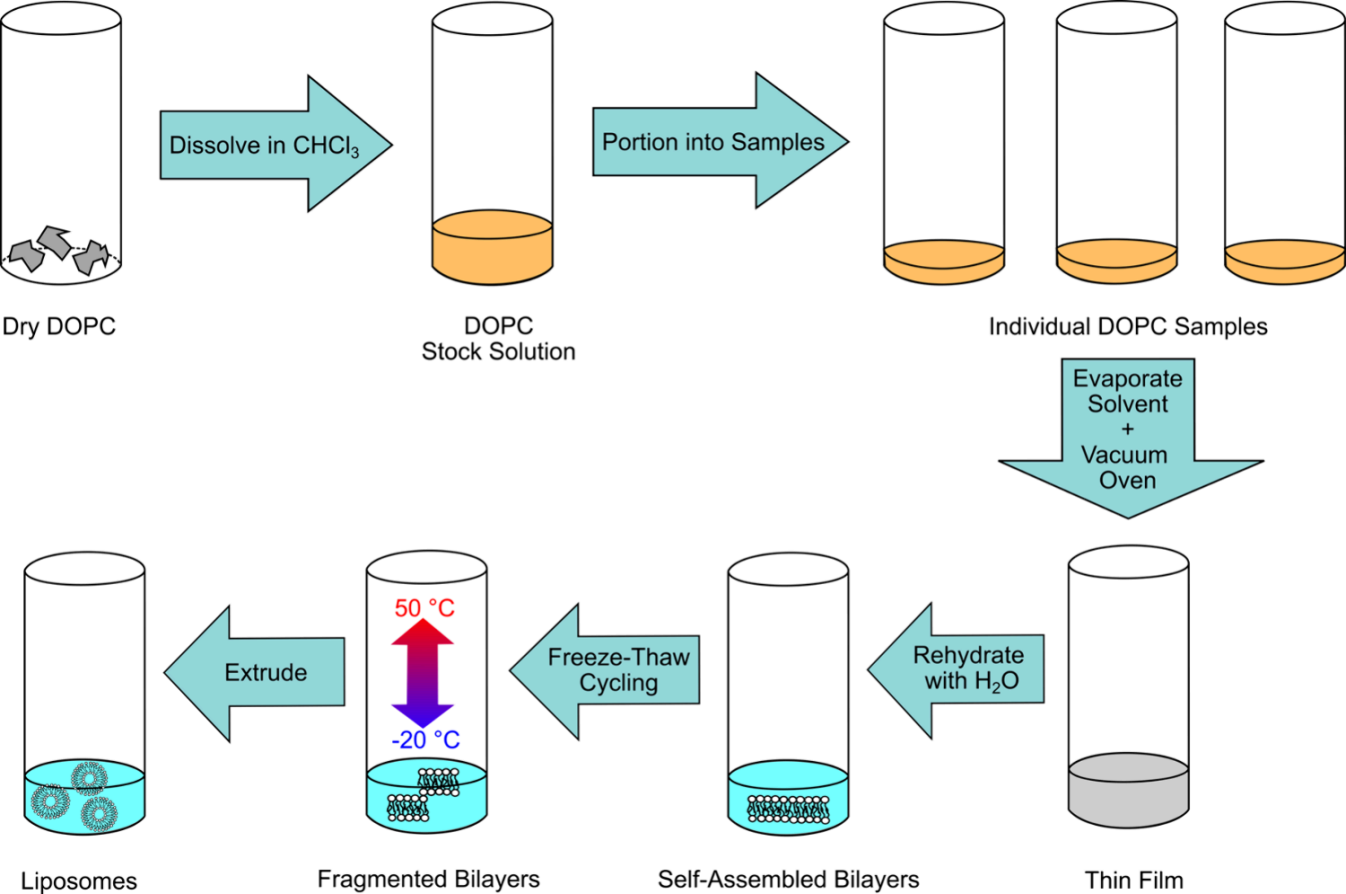
Schematic representation of the liposome sample preparation process.

1,2-Dioleoyl-*sn*-glycero-3-phosphocholine (DOPC) was purchased from the NOF American Corporation. The preparation procedure is adapted from an earlier work by this group.^22^ 54 mM DOPC stock solutions were prepared in chloroform. 0.5 mL aliquots of DOPC stock solutions were then evaporated under an N_2_ stream, and the resulting lipid cakes were stored under a vacuum overnight. The dried lipid cakes were then rehydrated with 2 mL of ultrapure water (18.2 MΩ/cm) and vortexed. Next, the samples were exposed to four freeze–thaw cycles. Each cycle consisted of 10 min at −20 °C in a freezer and 10 min at 50 °C on a hot plate. The samples were then extruded at room temperature (Figure 3). For the extrusion process, 100 nm polycarbonate membranes were used. All samples were extruded for an odd number of passes to remove any larger impurities present in the initial sample. These will be trapped behind the membrane, while the final pass will move the sample solution to the syringe that was initially empty. The extrusions were performed using freshly prepared samples at ambient temperature. About 5% of the lipid mass is lost via extrusion. This value decreases slightly as the number of passes increases (see Figure SI 2). Manual extrusion by the authors was found to average about 1200 μL/min (SI 6). Therefore, for the best comparison, the automatic extruder uses this system’s maximum flow rate of 800 μL/min.

### Dynamic Light Scattering (DLS)

Dynamic Light Scattering (DLS) was performed using a Malvern Zetasizer Nano ZS equipped with a 633 nm, 30 mW He–Ne laser at a scattering angle of θ = 173°, a temperature of 25 °C, and an equilibration time of 60 s. Hydrodynamic diameter, *D*_H_, and polydispersity, *PDI*, were determined. First, the dynamic correlation function was measured. From this, the Malvern Zetasizer Nano ZS instrumental software determined the *D*_H_ and *PDI* based on cumulants’ analysis using an exponential decay function.^32^ Each data point represents three averaged samples. Each sample has been measured three times, and the values were combined to form an average. Error bars shown for *D*_H_ or *PDI* represent the standard deviation.

## Automatic Extruder

### Project Groups and Timeline

The automatic extruder was designed for student researchers and features a simple setup. The project is assumed to fit within the limited number of hours a typical group of student researchers can spend in a research laboratory during a 12-weeks summer project. Once completed, the project could be further expanded upon. Readers are encouraged to upload software and hardware improvements, including technical drawings, to the authors’ GitHub archive intended to make the project freely available. The link can be found in the Supporting Information.

**Figure 4:**
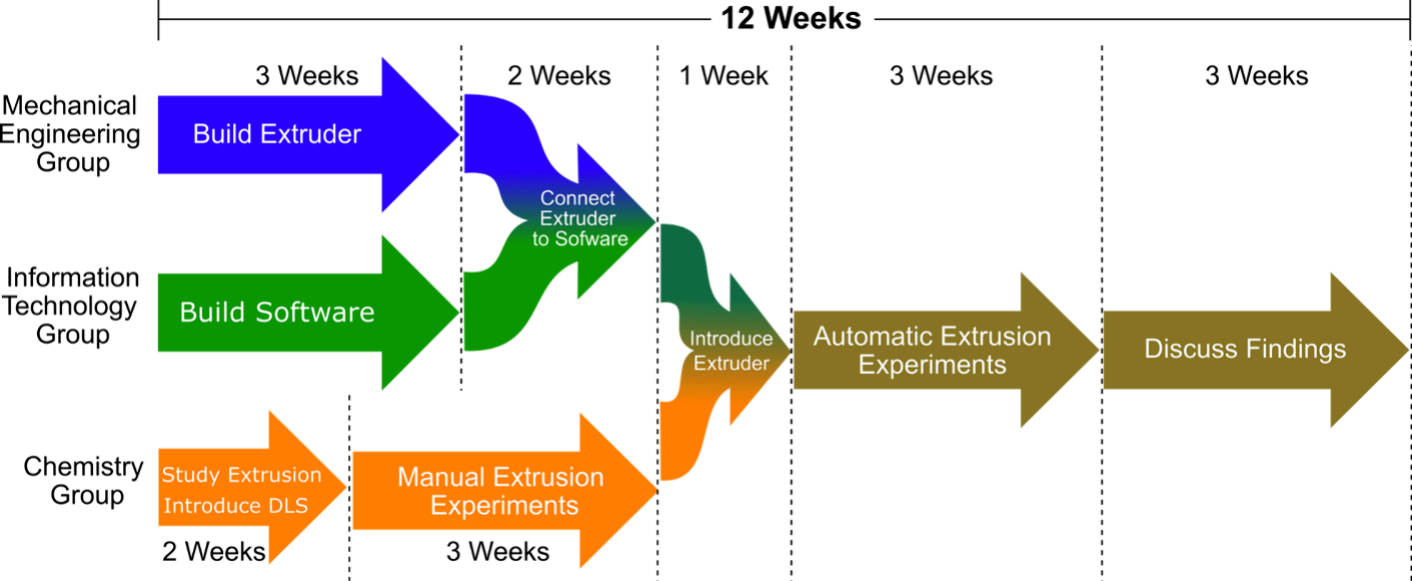
Suggested timeline to build the automatic extruder. Each group began by working on an individual project module. When these are completed, the modules are combined. The final project steps are performed by the students together.

The following is a suggestion on how to plan and build the automatic extruder. The initial setup of the project can be performed by three students or student groups. These can be assigned according to the students’ interests. The groups are a mechanical engineering group (ME), an information technology group (IT), and a chemistry group (CH). Each group will take about a semester to complete their task (see Figure 4).

The mechanical engineering group builds the automatic extruder and connects it to the computer. The information technology group sets up LabVIEW or similar software. The chemistry group performs experiments using the manual extrusion technique and then tests the automatic extruder once it is prepared. A suggested timeline is as follows: The ME and IT groups have 3 weeks to build the extruder and implement the software. The next 2 weeks are spent connecting the hardware to the computer. The CH group takes 2 weeks to familiarize themselves with the extrusion process and the dynamic light scattering technique. They then take 3 weeks to gather data using manual extrusion. After these 5 weeks, all three groups work together. In the first week, the ME and IT groups introduce the automatic extruder to the CH group. In the three following weeks, all groups perform the automatic extrusion experiments together and then take another 3 weeks to discuss and present their findings.

### Project Materials

**Figure 5:**
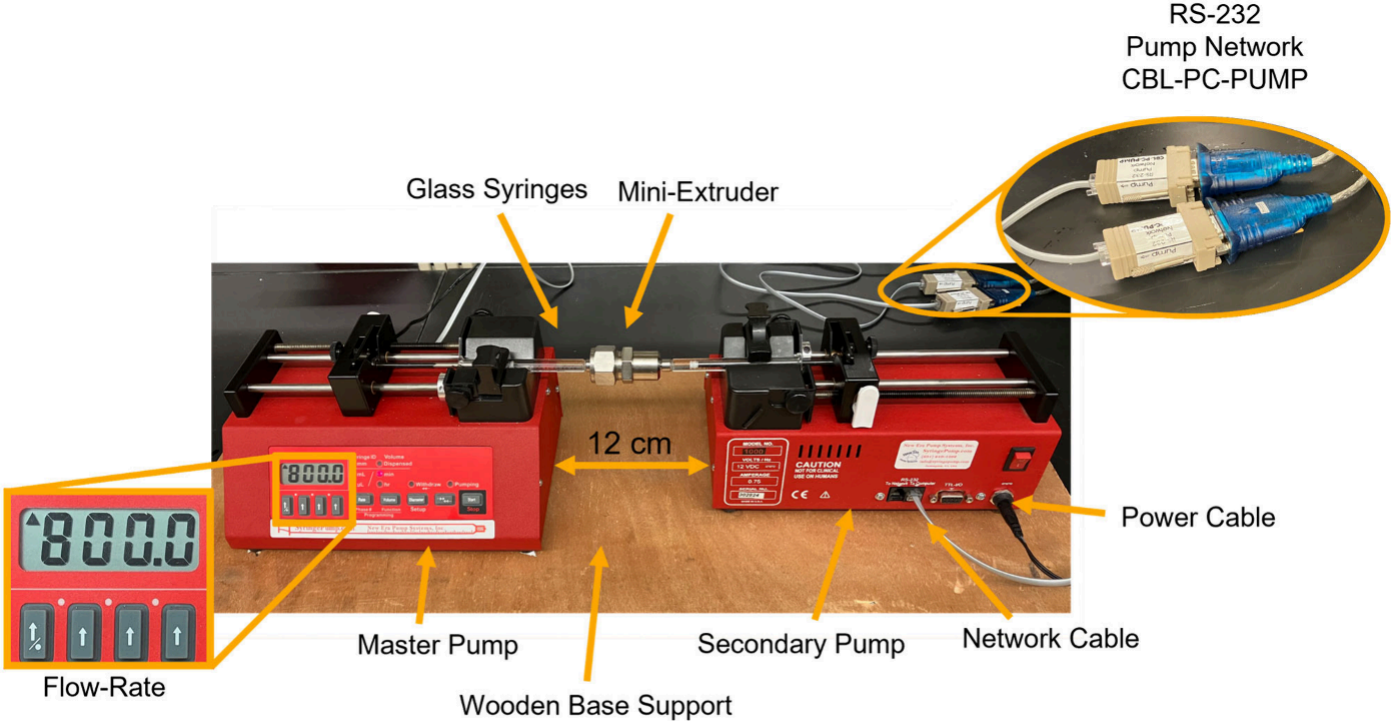
Parts are needed in order to set up the automatic extruder. Network and power cables are necessary for both pumps.

Every learning experience needs materials. Figure 5 shows the parts and components that must be available to set up the automatic extruder described in this project. In addition to what is displayed in the figure, a computer to run the software and sample preparation materials will also be needed. The New Era syringe pumps or Avanti Mini-Extruder used here can be substituted with hardware from other brands without impacting the extruder setup. Below is a list of the necessary components needed to run the project. All prices are from June 2024.

In order to run the project suggested here, a laboratory should have access to

- A dynamic light scattering instrument (used in macromolecular chemistry groups)
- A hand-extruder kit (∼$1000)^33^
- About 3 g of 1,2-Dioleoyl-*sn*-glycero-3-phosphocholine (DOPC) (∼$300,^34^ varies by supplier)
- 2 automatic syringe pumps ($815 each,^35^ may be available in synthetic chemistry group)
- A LabVIEW license (The LabVIEW community edition is free for noncommercial use.)
- Drivers for the specific syringe pumps (free, can be found on the project GitHub or the National Instruments Web site)^36^
- 2 1 mL gastight glass syringes with removable needles and an internal diameter of 4.61 mm. (Part of the mini-extruder kit available from Avanti Polar Lipids)
- 2 × 10 mm Filter supports (per 1 mL extruded solution)
- 1 × 0.1 μm Etched Polycarbonate Membrane (per 1 mL extruded solution)
- 1 computer with a monitor
- 2 RS-232 Pump Network (CBL–PC-PUMP) connector cables
- 2 Pump Power cables
- 2 network cables
- A hot plate (expected to be present in the lab)
- A vacuum oven or similar

### Syringe Pump Setup

The automatic extruder setup essentially consists of two automatic single syringe pumps (New Era Pump Systems, Inc.) operating a manual extruder. The pumps are fastened to a wooden board, with syringe holder blocks facing each other. A distance of 12 cm between the individual syringe pumps allows for one Avanti Mini Extruder, loaded with two 1 mL Hamilton gastight glass syringes, to be placed in the syringe pump system (see Figure 5). The two NE-50X syringe pumps are set up to communicate with one another to synchronize the pumping process. One pump is programmed as the “master” pump. Any configurable variable applied to the master pump is transmitted to the “secondary” syringe pump. Each pump is programmed with an address (typically 0 and 1, 0 being the master pump, and 1 being the follower pump) to be differentiated by the computer, which only communicates with the master pump. The two pumps communicate using a regular CAT-5 ethernet network cable connected to the back of the syringe pumps. An RS-232 to USB adapter connects the back of each pump (RS-232) to the computer (USB) running the LabVIEW program.

### Software Development

New Era Syringe Pumps can be connected to become a multipump network without third party programming.^37^ However, an automatic extruder built using this method depends on the pump’s manufacturer. Alternatively, custom software can be used. Several different programming tools, from machine or assembler code to high-level languages, are available today. Python, LabVIEW, and C++ are among the most common. The freely available Python has functionality similar to that of the commercial MATLAB. When deciding which tool to use, it is essential to identify the needs and circumstances of the project. Python is a character-based text file. It is equally suitable for calculation of display tasks and hardware control. C++ has seen much development and has large associated libraries. LabVIEW is a graphical language that uses drag-and-drop symbols with predefined functions to create code. Graphical user interfaces, available within the program, are used to control the data acquisition and storage hardware. In the case of the students, who worked on the project presented here, the ability to transfer a workflow diagram from the page into the LabVIEW block chart was critical. In this way, LabVIEW proved to be well-suited for beginners who have not been in contact with code before. Additionally, LabVIEW plug-and-play drivers for New Era Syringe Pumps are available from the National Instruments Web site.^36^ The finished program has been uploaded to the author’s GitHub page under the MIT License. This makes the software free for commercial and private use, to modify, and to distribute.

**Figure 6:**
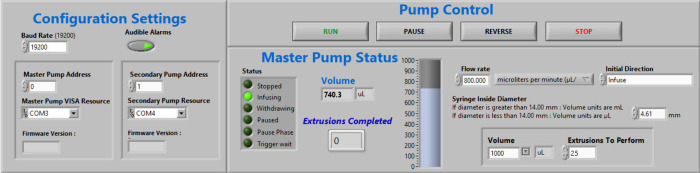
Graphical user interface for the LabVIEW software. “Configuration Settings” refer to the connection of the pumps to the computer, “Pump Control” contains tasks necessary start and stop the extrusion, and the “Master Pump Status” panel can be used to adjust extrusion parameters.

LabVIEW has the option to build an intuitive graphical user interface (Figure 6). For this project, the panel titled “Configuration Settings” contains parameters that control the connection of the extruder and the computer. The panel “Pump Control” can be used to start, stop, reverse, or pause the extrusion process. The panel “Master Pump Status” contains variables that can be changed according to the specific needs of the equipment. This includes options for flow rate, syringe diameter, the volume of the solution in the syringe, and the number of extrusions. Exchanging the syringe pumps is simple and can easily be accomplished by swapping the control module. The source code was uploaded to GitHub.

Future plans for the software development of the extruder presented here include the addition of a Raspberry PI. This would make the instrument smaller and more portable. However, the instrument currently available to the authors does not support the LabVIEW Program or the graphical user interface for syringe pumps. Further development may include changing to a programming language whose compiler produces faster and more portable code, which can then be implemented on the Raspberry PI.

## Testing

To validate the performance of the automatic extruder, it was compared to that of manual extrusion. The comparison focused on three factors: The number of passes through the extruder, the flow rate of the solution, and the effect of a stepwise change in flow rate. The differences in the hydrodynamic diameter, *D*_H_, and the polydispersity index, *PDI*, of the extruded material are evaluated.

### Influence of Number of Passes

**Figure 7:**
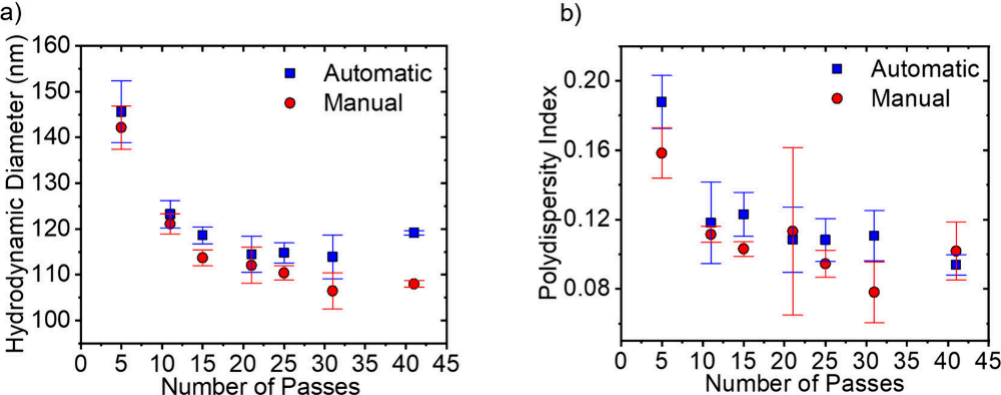
Influence of the number of passes for the automatic and manual extrusion data on the (a) *D*_H_ and (b) *PDI* of DOPC liposomes. All samples were extruded at ambient temperature (23 °C). The automatic extruder samples were extruded at a flow rate of 800 μL/min.

The number of passes through the membrane influences the size of the resulting vesicles.^38^ The more extrusion passes, the smaller the vesicles become. Eventually the size remains constant. In the students’ data, automatically and manually extruded samples decrease in *D*_H_ with an increasing number of passes, until a constant *D*_H_ is reached (see Figure 7a). The automatic extruder seems to produce liposomes with slightly larger diameters. The *PDI* of automatically and manually extruded samples decreases and then seems to remain constant at the same value, within error (see Figure 7b). These findings match available research.^39^

According to literature, large vesicles have a lower surface tension than small vesicles (see SI 4).^39^ The lower the vesicle radius, the larger the lysis tension needed to rupture the vesicle.^39^ The automatic extruder works based on a constant flow rate rather than a constant pressure. As fewer and fewer large vesicles remain in solution with each pass, the pressure built up by the plunger decreases. This happens because fewer vesicles are large enough to block the pores of the membrane. Once this pressure no longer reaches the larger lysis tension of the smaller vesicles, the vesicles no longer become smaller. During manual extrusion, the experimenter is hypothesized to naturally operate at constant pressure rather than at a constant flow rate. This causes the applied pressure to remain greater than the lysis tension for the vesicles for a longer time, leading to smaller vesicles. Sample 41 is considered an outlier.

The *PDI* values of the vesicles decrease as the number of extrusions increases (see Figure 7b). It decreases slightly faster for the manual extrusion than for automatic extrusion. At the maximum number of passes, both extrusion methods reach a similar *PDI*. *D*_H_ and *PDI* values for the automatic extrusion remained slightly above those achieved by manual extrusion. Given the reasoning above, manual extrusion may be able to keep a constant pressure for longer than automatic extrusion. This leads to a lower minimum diameter for the vesicles than is achievable by keeping the flow rate constant. This is because the constant flow rate causes a decrease in the pressure during automatic extrusion.

### Influence of Flow Rates

**Figure 8:**
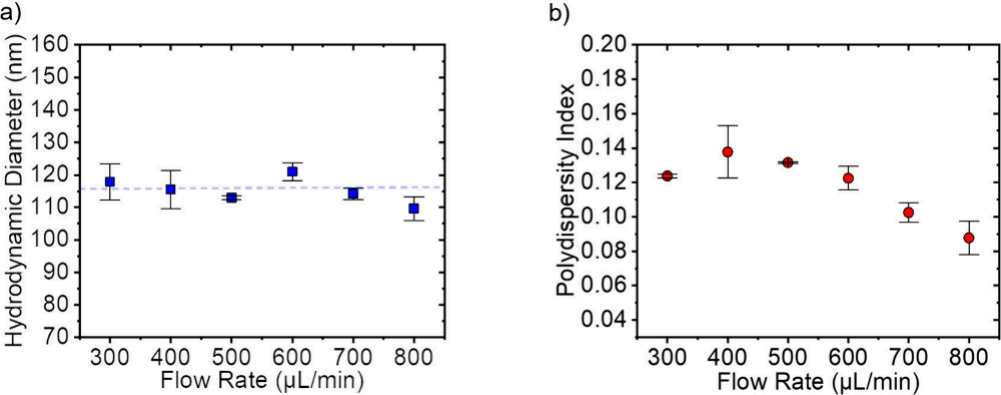
Influence of the flow rate on (a) *D*_H_ and (b) *PDI* of the DOPC liposomes extruded automatically with varying flow rates. The horizontal blue line marks the average liposome diameter.

Flow rates are the main variables that can be controlled using automatic extrusion. The flow rates are investigated using only the automatic extruder, as constant flow rates are challenging for a human experimenter to achieve. Each flow rate sample was extruded for a total of 21 passes. In the previous section, this value was found to be the number of passes at which the vesicle *D*_H_ remains constant. Figure 8 shows the *D*_H_ and *PDI* values of the extruded liposomes as a function of the flow rate used for their extrusion. There is no dependence of *D*_H_ on the flow rate (see Figure 8a) and a slight dependence of *PDI* (see Figure 8b). The highest flow rate of 800 μL/min shows a *PDI* of around 0.09, while a 400 μL/min flow rate resulted in around 0.14.

Increasing the automatic extruder’s flow rate does not affect the minimum achievable vesicle diameter. This agrees with findings from the literature, where a doubled flow rate did not affect the size of the vesicles.^24^ The *PDI*, on the other hand, clearly decreased with an increasing flow rate (see Figure 8). This is counter to a result by Frisken et al., who found that *PDI* was not connected to any of the extrusion process variables. They suggest that this may be because extrusion could be considered a type of fragmentation process, for which these wide size distributions would be expected.^24^

### Influence of a Stepwise Flow Rate Adjustment

The resistance of the lipid solution decreases as it repeatedly passes through the membrane.^24,40^ In an effort to mimic the behavior of a human experimenter performing manual extrusion, the flow rate of the automatic extruder was increased in a stepwise manner during the extrusion process. The authors speculate that unlike automatic extrusion, manual extrusion results in a constant pressure because the researcher unconsciously adjusts the flow rate during the extrusion process. The syringe pump used here cannot independently change the extrusion pressure.

**Figure 9:**
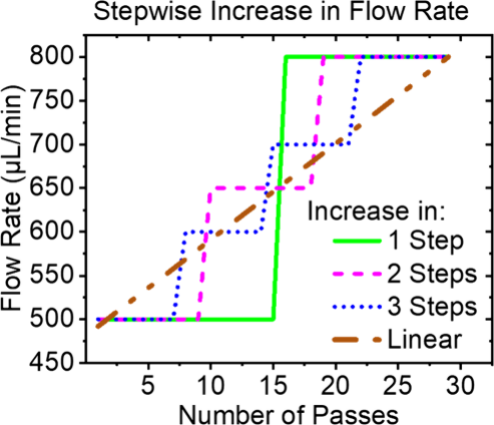
Flow rate profiles for 1 Step to Linear samples are displayed graphically. In the flow rate profile 1 Step, the sample was extruded for 15 passes at 500 μL followed by 14 passes at 800 μL. Two Steps was extruded for 9 passes at 500 μL, followed by 9 passes at 650 μL/min, and finally, 11 passes at 800 μL/min. Three Steps was extruded for 7, 7, 7, and 8 passes at 500 μL, 600 μL, 700 μL, and 800 μL respectively. Sample Linear was continuously increased by 11 μL with every pass.

Since, according to Darcy’s law (see equation S1), flow rate is proportional to the pressure across the pore entrance, it was incrementally increased over the extrusion process. The flow rate was increased from 500 to 800 μL/min in a different number of steps over a total of 29 passes. The flow rate profiles can be seen in Table 1 and Figure 9. For a further explanation of the number of passes at each flow rate, see SI 7.

**Table 1:** Flow rates and number of passes for each number of flow rate changes (P2-4) used for stepwise flow-rate studies of the automatic extruder

1 Step		2 Steps		3 Steps	
Flow Rate (μL/min)	Passes (Nr.)	Flow Rates (μL/min)	Passes (Nr.)	Flow Rates (μL/min)	Passes (Nr.)
500	15	500	9	500	7
800	14	650	9	600	7
		800	11	700	7
				800	8

**Figure 10:**
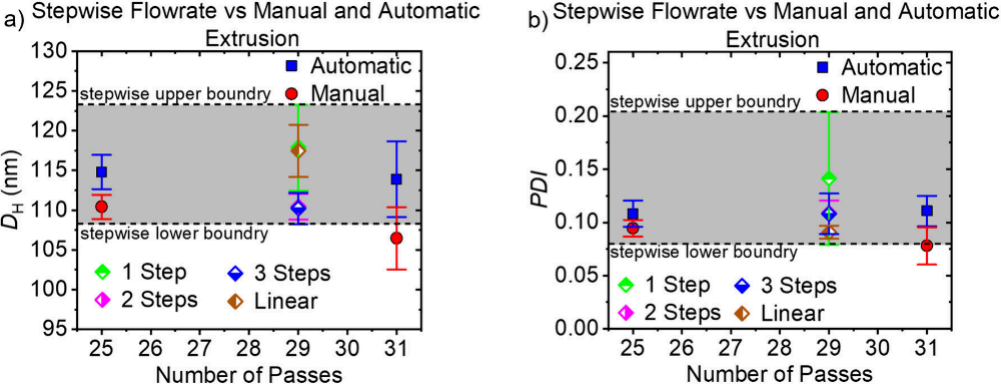
Results of the stepwise flow rate trials as well as the automatic and manual values for 25 and 31 passes for *D*_H_ (a) and *PDI* (b) of the DOPC liposomes. One Step is shown in green, 2 Steps in pink, 3 Steps in blue, and linear in orange.

The results of the stepwise increase in the flow rate are shown in Figure 10. For comparison to *D*_H_ and *PDI* for each flow rate profile, automatic and manual values for 25 and 31 passes are shown. The gray background marks the upper and lower boundary for *D*_H_ and *PDI* of all flow rate profiles including their error bars. Within experimental accuracy, changing the flow rates in a stepwise manner had no effect on the *D*_H_ and *PDI* of the liposomes. The *D*_H_ and *PDI* values of automatic extrusion achieved with 25 and 31 passes fall completely within the gray zone. The manual extrusion values do not.

The stepwise increase in flow rate in order to mimic the constant pressure during manual extrusion resulted in *D*_H_ values that are similar to those obtained by automatic extrusion. In addition, the values seem to be independent of the flow rate profile (see Figure 10).

## Perspectives

The project described here supplies instructions on how to build an open-source automatic extruder. The building specifications can be adapted to the student’s backgrounds, interests, and experimental needs. Many of the components may already be available from the research group or the department. Once present, the automatic extruder can be used to quickly produce reliable results, independent of the students’ current level of training. Using an automatic extruder limits the number of changing variables. The project can serve as an introduction to interdisciplinary work, first contact with a vital self-assembly process, and help in understanding an industrial method on the small scale. It encourages cooperation, creativity, and critical thinking. The preparation of liposomes can be a valuable tool to connect in-lab experiments to real-life applications. Comparing manual to automatic extrusion reliably illustrates liposome preparation theory, specifically the pressure dependence, found in the literature. Manual extrusion allows students to learn about the importance of changing a single variable when it comes to a scientific outcome. The flow-rate-based extrusion system cannot be adjusted to mimic manual extrusion by adjusting the flow rate throughout the process. However, smaller vesicles may only be necessary for specific drug delivery applications.^28^ The extruder’s value becomes apparent when applied to projects necessitating large throughput where a slightly larger vesicle diameter is acceptable.

To further simplify the setup and make it more suitable for an undergraduate laboratory environment, a Raspberry PI or tablet could be implemented to make the operation more intuitive. Additionally, the syringe pumps may be adapted to operate in a pressure-sensitive mode. This may lead to greater control over vesicle diameters and allow for further discussion of the theory involved in vesicle preparation by extrusion.

## Data Availability

GitHub Link https://github.com/gschneidergroup/Simplistic-Automatic-Extruder

## Supporting Information Available

LabVIEW workflow file (PDF)
LabVIEW workflow file (DOCX)
Block Diagram (PDF)
