# Supplementary material for: Building a Simplistic Automatic Extruder: Instrument Development Opportunities for the Laboratory
Source: J Chem Educ. 2024 Aug 1;101(8):3292–300. doi: 10.1021/acs.jchemed.4c00287 (PMC11327960; doi:10.1021/acs.jchemed.4c00287)
Supplement: Supplementary file 1 — ed4c00287_si_001.pdf [file ed4c00287_si_001.pdf]

Building a Simplistic Automatic Extruder: Instrument Development Opportunities for the Laboratory

Stefanie Klisch,[1] Dylan Gilbert,[2] Emma Breaux,[1] Aliyah Dalier,[2] Sudipta Gupta,[1] Bruno Jakobi,[1] Gerald J. Schneider[1,3]

1 Department of Chemistry, Louisiana State University, Baton Rouge, LA 70803

2 Department of Chemistry and Physics Southeastern Louisiana University, Hammond, LA 70402

3 Department of Physics & Astronomy, Louisiana State University, Baton Rouge, LA 70803

Corresponding author: gjschneider@lsu.edu

SI 1: INFLUENCE OF WATER ON LIPOSOMES: DEIONIZED VS ULTRAPURE

The influence of the type of water of water on the liposomes' hydrodynamic diameter (D_H) and polydispersity (*PDI*) was tested. Three additional samples of liposomes were prepared using deionized water from the wall faucet in the laboratory. These were compared with the results of liposomes prepared in ultrapure water (18.2 MΩ/cm) of the main text. For the liposomes in deionized water the same preparation using the automatic extruder (flow rate of 800 µL/min through a 100 nm polycarbonate membrane extruded for 21 passes) was chosen. Figure S1 shows that there is no change in the diameter due to the change in water source within the accuracy of the experiment.

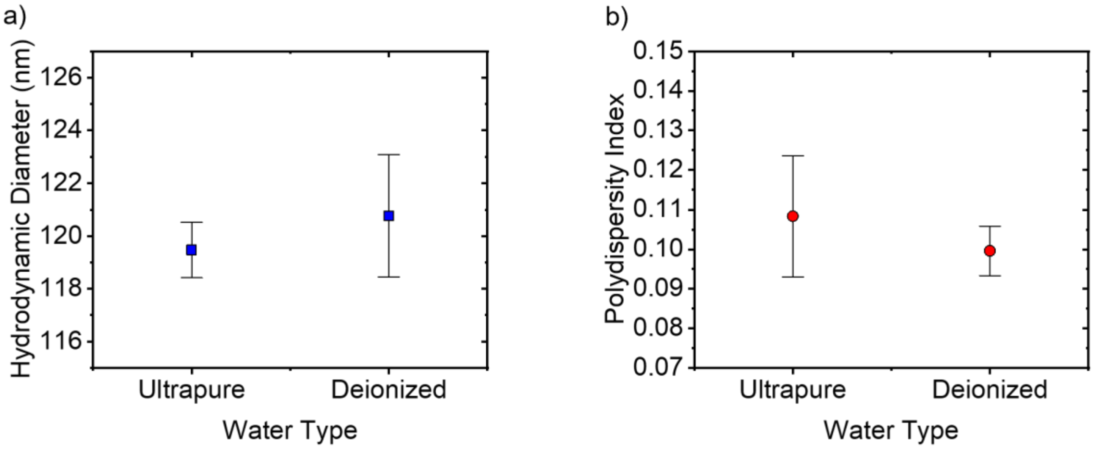

Figure S 1: Liposome a) hydrodynamic diameter (D_H) and b) polydispersity (*PDI*) for samples prepared using deionized water from the laboratory faucet and using a Barnstead Smart2Pure filtration system operating at a resistivity of 18.2MΩ/cm.

SI 2: SAMPLE LOSS DURING EXTRUSION

In order to study a possible decrease in lipid concentration due to extrusion, the amount of sample left in the filter supports and membrane after extrusion was quantified. The mass of the polycarbonate membrane and the two filter supports was recorded before and after extrusion of the samples. Three different numbers of extrusions 5, 11, and 21 passes were tested (see Figure S2). For each number of passes, three samples were prepared. Each was extruded using the automatic extruder at 800 µL/min through a 100 nm polycarbonate membrane. The used polycarbonate membranes and filter supports were then dried overnight, and their mass recorded the next day. All samples showed an average loss of about 6% of

sample mass remaining in the polycarbonate membrane and filter supports after extrusion. While the average value decreases, the values are within each other's error bars.

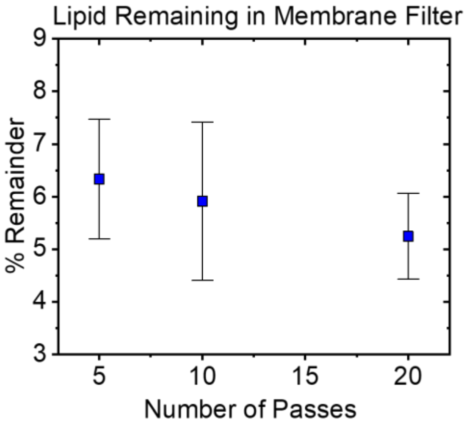

Figure S 2: Lipid remaining in the polycarbonate membrane after automatic extrusion at 800 μL/min.

SI 3: GITHUB LINK

The GitHub archive containing the LabVIEW workflow file, licensing information, drivers, and additional information can be found by following this LINK.

SI 4: FLUID DYNAMICS AND PARTICLE SIZE IN VESICLE FORMATION

Models describing the process of extrusion through a membrane are available in the literature. These models generally assume laminar flow (Q) within the pore.[1, 2] Clerc et al. proposed that a velocity gradient within the pore causes shear of the individual lipid lamellae.[1] The fluid near the center of the pore moves faster than near the edge of the pore. As large vesicles entering the pore are bent to a curvature higher than what their bilayer can tolerate, they break and re-seal into smaller vesicles. Since the movement of the solution within the pore is laminar, Darcy's Law has been widely used to describe the extrusion process.[1-3] The flow rate shows a linear dependence on the pressure difference between the entrance and exit of the pore.[3, 4] Equation 1 shows the corresponding function.

$$\eta Q = K\left(\frac{\Delta P}{\Delta L}\right) \tag{1}$$

Here, Q is the rate of laminar flow as it passes through a cylindrical pore. Over a distance ΔL, there is an applied pressure difference of ΔP. Darcy's Law describes the behavior of a homogenous fluid. Applied to solutions containing vesicles, it holds true for extrusions at high pressures,[2, 4] or at low concentrations and low pressures.[3] In both cases, the volume flow rate is proportional to the applied pressure. The factor K in its original form describes the geometrical form of the pore. However, given that the fluid contains vesicles that need to be pushed through the pore, Bruinsma[2] converted it into an effective permeability K_{eff} which is given by equation 2:

$$K_{eff} = \frac{N\pi R_p^4}{8+0.233(nL^*)\left(\frac{L^*}{R}\right)} \tag{2}$$

Where N is the number of pores in the membrane, R_p is the average radius of the pores, n is the number of vesicles in a pore of length L^*, and R is an effective radius of the pore that is dependent on the velocity of the fluid moving through this pore. As the fluid moves through the pore, a lubrication layer forms along the side of the channel which increases in thickness as the velocity of the fluid increases.[2] This phenomenon is given by $R = R_p\text{-}h^*(\upsilon)$ where h^* is the thickness of the lubrication layer and υ the velocity of the fluid.

Based on the polydispersity of the extruded vesicles, the mechanism by which the vesicles are formed is pressure- rather than flow rate-dependent.[3, 5] However, flow rate has

also been observed to be linked to the size of the extruded vesicles.[4] Studies of emulsions and lamellar phases exposed to shear by Diat[6] and Mason and Bibette[7] showed increased shear leading to smaller droplets. Hunter et al.[4] explain this effect via the layer of lubrication that forms on the inside of the pore as the solution is extruded. The higher the flow rate, the thicker this lubrication layer, the smaller the effective pore size. It is well documented, that the main factor determining the size of the extruded vesicles is the pore size.[8]

The diameter of the resulting vesicle, especially during the first extrusion pass, is determined when the large vesicle enters the pores of the membrane.[3] As more and more vesicles reach the necessary size to easily pass through the pores of the membrane, the pressure needed to press the vesicles through the membrane continually decreases.[3] The lower the extrusion pressure, the larger the resulting vesicle.[3] In reverse, studies using extruders featuring constant extrusion pressures find an increase in flow rate which eventually plateaus once all vesicles are small enough to easily pass through the membrane pores.[4]

The pressure needed to for a vesicle to move through the pore is equal to the lysis tension of this vesicle because the passage through the pore causes the vesicle to rupture.[4] The Laplace relation (equation 3) gives the pressure difference necessary to break the surface tension of an interface, depending on the curvature of that interface.[4, 9]

$$\Delta P = 2\gamma H \tag{3}$$

All variables apply to the interface: ΔP is the pressure difference across the interface, γ is the surface tension, and is the mean curvature. Based on equation 3, Hunter et al. expanded the concept to take into account the radii of the vesicle being extruded and of the membrane pore.[4] This new function is shown in equation 4.

$$P_1 - P_0 = 2\gamma \left[\frac{1}{R_p} - \frac{1}{R_o}\right] \approx 2\gamma \left(\frac{1}{R_p}\right) \tag{4}$$

Here, P_1 is the pressure applied to the solution, P_0 is the atmospheric pressure, R_p is the radius of a vesicle small enough to pass through the pore, and R_o is the radius of the original larger vesicle being pushed against the pore entrance.

SI 4: LABVIEW PROGRAM SETUP

The LabVIEW workflow file available in the supplementary information shows LabVIEW's block chart for this project. In all LabVIEW applications, there is a starting set of variables collected from a resource. In this case, the initial resource is a driver software downloaded from the National Instruments page for the syringe pumps. These are also available on the authors' GitHub page. Once these variables are ready to be used, they can be imported into one of the program's SubVIs. SubVIs are small repeatable pieces of LabVIEW code within a larger LabVIEW program, the VI or Virtual Instrument.[10] They allow the software to become more organized and reduce software development time by streamlining the code in a visual manner. This is especially helpful because repeated pieces of code are easy to notice.[10] Referring to the workflow file provided in the supplementary information: Section A shows a series of SubVIs (pieces of code enclosed in a grey barrier) whose objectives are to set presets to the two syringe pumps before the operation has started. Section B shows the repeated piece of code during the run time of the software. This is LabVIEW's equivalent translation to a "For Loop" and behaves similarly to other software languages. Information from within this loop status is constantly sent to the front panel to assure the user that the instrument is operating correctly throughout the duration of the run time. Section C is the following loop primarily checking for the additional commands from the user during the run time of the instrument. This piece of the code also handles any error codes that may arise throughout the runtime and shuts down the instrument in the case of an error code. On the left beginning side of the block chart, the initialization of the syringe pumps and configuration settings are sent to the pump. Following this chain of commands, a "For" command block is presented to run the given configuration until it is complete, or the stop command is pressed on the front panel of the LabVIEW software.

The SubVIs within the LabVIEW block chart are designed in a way to be interchangeable with different pumps and their respective drivers. The drivers presented with the software will work with a large set of pump instruments.

SI 5: LABVIEW GRAPHICAL USER INTERFACE

A graphical user interface (GUI) can be created using LabVIEW (see Figure S 3). The essential part of the GUI is the Pump Control panel that allows the user to select important parameters, such a flow rate, solution volume, and number of extrusions to perform as well as the inner diameter of the syringes used and the initial direction of extrusion. The hardware limits on the syringe pump are stored in a separate configuration file. The flow rate set through the software is displayed in the display on the user interface of the master pump.

This version of the software assumes the two-syringe pump setup illustrated by the Configuration Settings panel (left) that shows a master and a secondary pump. To connect it with the computer a USB to serial adapter is installed and the ports COM3 and COM4 assigned to the respective USB ports. Hence, the PC with Windows 11 operating system and the syringe pumps are connected by a serial (RS-232) interface that communicates with a baud rate of 19200 baud.

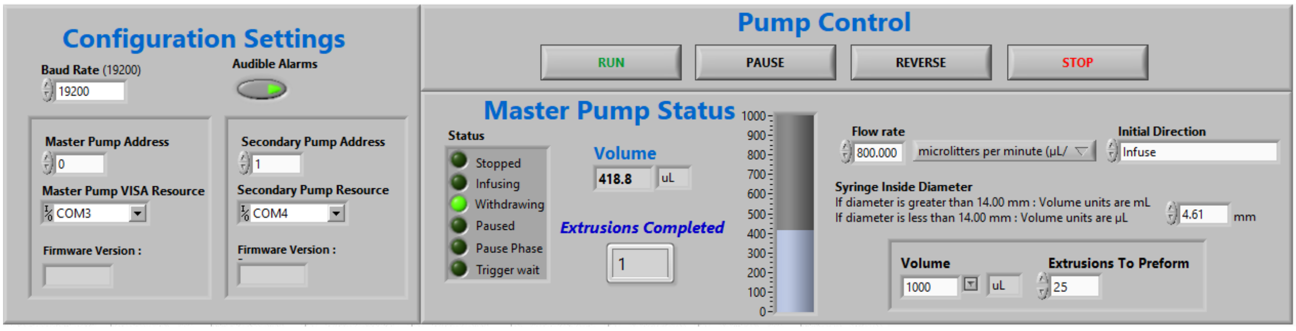

Figure S 3: Screenshot of the LabVIEW control panel for the two-pump automatic extruder software. The configuration settings on the right show the connection to the computer while the pump control is used to set parameters for the extrusion that will be executed. The link to the GitHub can be found HERE.

SI 6: MANUAL EXTRUSION FLOWR RATE

Table S 1 shows the time it takes a manual extruder for one extrusion pass with a volume of 1 mL.

Table S 1: Time taken by manual extruder to extrude 1 mL of fluid.

Trial	t/s
1	48
2	57
3	50

The average flow rate was found to be 1176 µL/min.

SI 6: MANUAL EXTRUSION BY DIFFERENT STUDENTS

Two trained students independently performed manual extrusions to compare the liposomes' D_H and *PDI* related to different experimenters (Figure S 4). With the exception of Figure S 4, all values marked as "manual extrusion" include samples prepared by students 2 and 3. Data by student 1 was taken from an earlier work[11] by the authors' group. The values were not included in the averages presented in the other figures because different numbers of passes were used. Figure S 4 shows the D_H and *PDI* of extruded samples as a function of the number of extrusions using the automatic extruder. Data by the individual students is marked with different colors and symbols. For student 1, standard deviations fell within the symbol size. Error bars were therefore omitted. For all students, the D_H fell below the value achieved using the automatic extruder (see Figure S 4a). Except for the 41 passes value, this is also the case for the *PDI* (see Figure S 4b). This result corroborates the earlier suggestion that the natural constant pressure during manual extrusion resulted in a lower minimum diameter than could be achieved by the automatic extrusion.

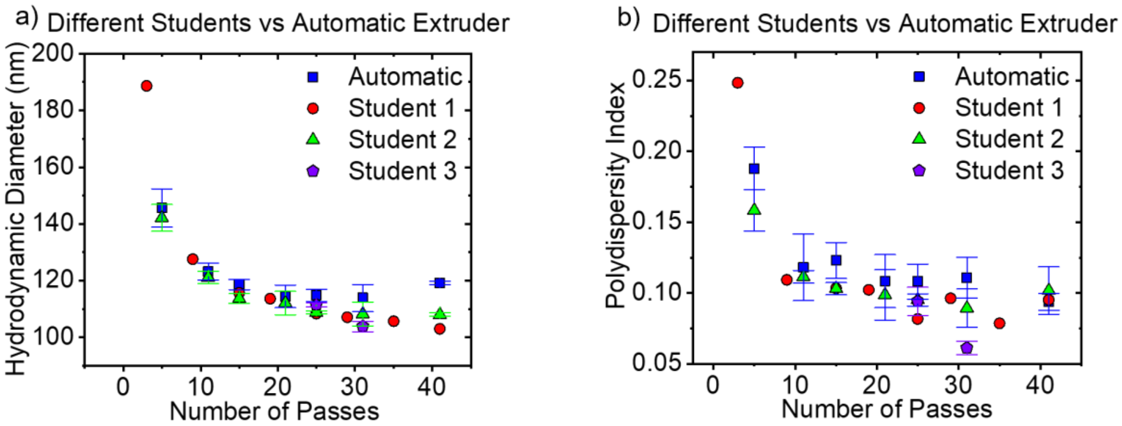

Figure S 4: Comparison of data extruded by different students. For data by student 1, the standard deviation was within symbol size. The data was digitized from an earlier work.[11] a) shows D_H as a function of the number of passes and b) the *PDI* as a function of the number of passes. The flow rate of the automatic extruder was 800 µL/min. All extrusions were performed at ambient temperature (23 °C).

SI 7: EXPLAINATION OF FLOW RATE PROFILES

The value of 29 was chosen because it is close to the previously used value of 31, an odd number of passes (see sample preparation), and allows for roughly equal numbers of passes at each flow rate. This created a set of different flow rate profiles (Figure 9). For the first sample (1 Step), 15 extrusion passes were performed at a flow rate of 500 µL/min,

followed by 14 extrusion passes at 800 µL/min. The 2 Steps sample was extruded for 9 passes at a flow rate of 500 µL, followed by 9 passes at 650 µL/min and finally, 11 passes at 800 µL/min. The 3 Steps sample was extruded for 7, 7, 7, and 8 passes at 500 µL, 600 µL, 700 µL, and 800 µL respectively. Finally, an additional flow rate sample (Linear) was added, for which the flow rate was continuously increased by 11 µL/min with every pass.

SI 8: DISCUSSION OF OUTLIER VALUE

The fact that the pressure decreases during the process of automatic extrusion may be the reason for the stagnation in the vesicle diameter at larger values than for manual extrusion (see Figure 7a). The 41 passes diameter value falls just outside the 35 passes diameter value when considering error bars. In contrast, the previous two values fell continuously within each other's errors. This may suggest that the 41 passes value should be considered an outlier and excluded from the analysis. Excluding the 41 passes diameter value would cause the diameter value of the automatic extrusion to remain constant at slightly lower diameter values. It would also mirror the manual extrusion more closely.

REFERENCES:

(1) Clerc, S. G.; Thompson, T. E. A possible Mechanism for Vesicle formation by Extrusion. *Biophys. J.* **1994**, *67*, 475-477.
(2) Bruinsma, R. Rheology and shape transitions of vesicles under capillary flow. *Physica A* **1996**, *234* (1-2), 249-270. DOI: 10.1016/S0378-4371(96)00358-5.
(3) Frisken, B. J.; Asman, C.; Patty, P. J. Studies of Vesicle Extrusion. *Langmuir* **2000**, *16* (1), 928-933. DOI: 10.1021/la9905113.
(4) Hunter, D. G.; Frisken, B. J. Effect of extrusion pressure and lipid properties on the size and polydispersity of lipid vesicles. *Biophys. J.* **1998**, *74* (6), 2996-3002. DOI: 10.1016/S0006-3495(98)78006-3.
(5) Patty, P. J.; Frisken, B. J. The pressure-dependence of the size of extruded vesicles. *Biophys. J.* **2003**, *85* (2), 996-1004. DOI: 10.1016/S0006-3495(03)74538-X.
(6) Diat, O.; Roux, D.; Nallet, F. Effect of shera on a lyotropic lamellar phase. *J. Phys. II* **1993**, *3*, 1427-1452. DOI: 10.1051/jp2:1993211.
(7) Mason, T. G.; Bibette, J. Emulsification in Viscoelastic Media. *Phys. Rev. Lett.* **1996**, *77* (16), 3481-3484. DOI: 10.1103/PhysRevLett.77.3481.
(8) Hope, M. J.; Bally, M. B.; Webb, G.; Cullis, P. R. Production of large unilamellar vesicles by a rapid extrusion procedure: characterization of size distribution, trapped volume and ability to maintain a membrane potential. *Biochim. Biophys. Act* **1985**, *812* (1), 55-65. DOI: 10.1016/0005-2736(85)90521-8. Mayer, L. D.; Hope, M. J.; Cullis, P. R.; Janoff, A. S. Solute distributions and trapping efficiencies observed in freeze-thawed multilamellar vesicles. *Biochim. Biophys. Act* **1985**, *817* (1), 193-196. DOI: 10.1016/0005-2736(85)90084-7. Mayer,

L. D.; Hope, M. J.; Cullis, P. R. Vesicles of variable sizes produced by a rapid extrusion procedure. *Biochim. Biophys. Act* **1986**, *858* (1), 161-168. DOI: 10.1016/0005-2736(86)90302-0. Hope, M. J.; Bally, M. B.; Mayer, L. D.; Janoff, A. S.; Cullis, P. R. Generation of Multilamellar and Unilamellar Phospholipid-Vesicles. *Chem. Phys. Lipids* **1986**, *40* (2-4), 89-107. DOI: Doi 10.1016/0009-3084(86)90065-4.
(9) Landau, L. D.; Lifshitz, E. M. *Fluid Mechanics*; Pergamon Press, 1987.
(10) Instruments, N. *Create and Configure as LabVIEW SubVI*. 2024. https://knowledge.ni.com/KnowledgeArticleDetails?id=kA03q000000YK4VCAW&l=en-US (accessed Jun 2024).
(11) De Mel, J. U. Structure and Dynamics of Phospholipid Vesicles and the Dependence on Nanoscale Interactions with Molecles of varying Complexity. Louisiana State University, Baton Rouge, LA, 2020.
