# Supplementary figures and images for: Building a Simplistic Automatic Extruder: Instrument Development Opportunities for the Laboratory
Source: J Chem Educ. 2024 Aug 1;101(8):3292–300. doi: 10.1021/acs.jchemed.4c00287 (PMC11327960; doi:10.1021/acs.jchemed.4c00287)

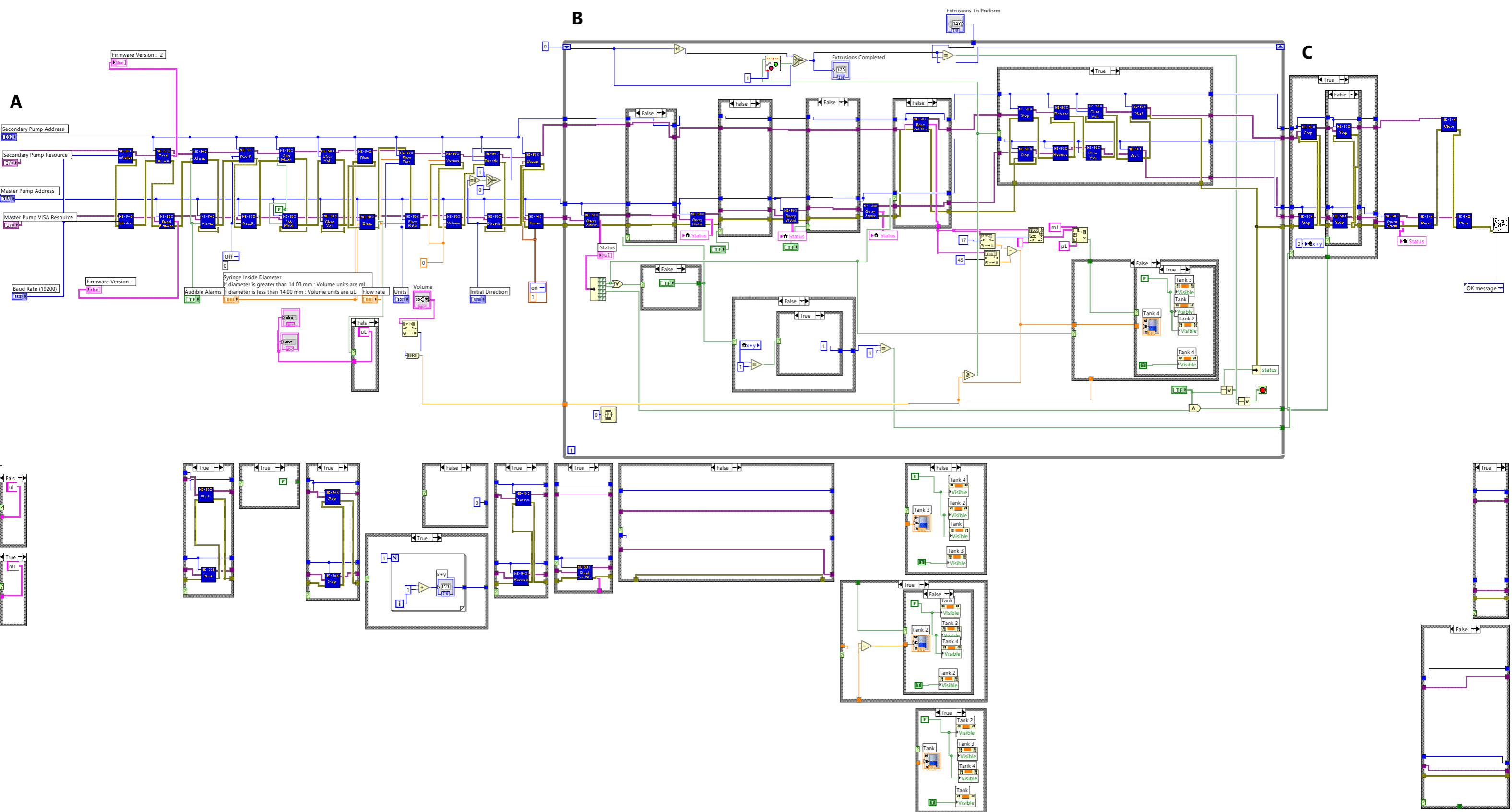

Supplement: Supplementary file 3 — ed4c00287_si_003.pdf [file ed4c00287_si_003.pdf]
